# Narrower Mesh Arm Width Is an Independent Predictor of Prolapse Recurrence After Transvaginal Mesh Surgery Using ORIHIME Mesh

**DOI:** 10.7759/cureus.81882

**Published:** 2025-04-08

**Authors:** Kenji Kuroda, Koetsu Hamamoto, Kazuki Kawamura, Hiroaki Kobayashi, Keiichi Ito

**Affiliations:** 1 Department of Urology, National Defense Medical College, Tokorozawa, JPN

**Keywords:** female urology, mesh arm width, pelvic organ prolapse, pelvic organ prolapse stage, transvaginal mesh surgery

## Abstract

Introduction: Transvaginal mesh surgery (TVM) is a less invasive and time-efficient surgical technique for the treatment of pelvic organ prolapse (POP). However, POP recurrence is a concern in patients treated with TVM using the ORIHIME (Kono Seisakusho, Japan, Tokyo) mesh. In this study, we investigated the influence of mesh-related factors on POP recurrence after TVM using ORIHIME.

Methods: The study enrolled 104 patients who underwent TVM at our hospital between July 2019 and March 2024. The Pearson chi-squared test, multiple logistic regression analysis, and Cox proportional hazards model were used to identify independent predictors of prolapse recurrence.

Results: Among preoperative and intraoperative factors, POP stage 4 and mesh arm width < 6 cm were significantly associated with prolapse recurrence (both p <0.05). On multiple logistic regression analysis, only the mesh arm width < 6 cm was a significant predictor of recurrence (p = 0.0077). Additionally, in the multivariate Cox proportional hazards model, only the mesh arm width < 6 cm was an independent predictor of shorter time to prolapse recurrence (hazard ratio, 8.39; 95% confidence interval, 1.70 - 41.35; p = 0.0089).

Conclusion: Narrow mesh arm width can contribute to POP recurrence after TVM using ORIHIME. Applying wider mesh arms can help minimize the POP recurrence rate.

## Introduction

Pelvic organ prolapse (POP) is a highly prevalent condition affecting approximately 50% of parous women, with its frequency increasing with age [1]. In their lifetime, ≥ 10% of women will require at least one pelvic reconstruction procedure [2]. The treatment options for POP vary depending on the severity of the condition, comorbidities, and individual conditions related to sexual and reproductive health.

Laparoscopic or robot-assisted sacrocolpopexy (LSC or RSC) and transvaginal mesh surgery (TVM) are representative examples of abdominal or vaginal approaches to treat POP surgically using mesh. RSC is considered to be the best treatment for apical prolapse owing to its high success rate and low recurrence rate; however, it is associated with a greater cost and entails a longer surgical time [3,4]. Conversely, TVM offers a less invasive, time-efficient alternative but is associated with notable adverse effects, including mesh exposure and de novo lower urinary tract symptoms. However, based on our previous studies and existing literature, TVM appears to be an excellent technique with favorable outcomes that are not inferior to sacrocolpopexy [5-8].

In Japan, mesh surgery for POP is not prohibited. Since kits for TVM are not sold in Japan, the surgery is performed using the so-called self-cut mesh, which is cut to fit a stencil paper. Currently, Japan’s sole approved mesh product for TVM is ORIHIME (Kono Seisakusho, Japan, Tokyo), composed of polytetrafluoroethylene (PTFE), a fluoropolymer (carbon fluoride resin) composed entirely of fluorine and carbon atoms. PTFE boasts exceptional properties, including a low disintegration rate, low tissue reaction rate, and chemical stability. It has been extensively used in various medical applications, such as artificial blood vessels, sutures, cardiac repair patches, and hernia repair mesh [9,10].

PTFE has been shown to cause less tissue inflammation than polypropylene (PP) mesh, suggesting its potential as a safer in vivo alternative [11]. During our experience with TVM surgery using ORIHIME, we observed that the mesh possesses desirable characteristics, including thinness, lightness, and strength. However, we also noted a tendency for the ORIHIME mesh to slide off the surrounding tissue. In response, we gradually widened the mesh arm that penetrates the sacrospinous ligament [5].

In the present study, we analyzed the data of patients with POP who underwent TVM at our institution and investigated the potential influence of mesh arm width on POP recurrence after TVM using ORIHIME.

## Materials and methods

Patients

The medical records of 104 patients who underwent TVM between July 2019 and March 2024 were retrospectively reviewed. The surgical indication was POP of stage ≥ 2 associated with symptoms (such as a sensation of vaginal protrusion) or hydronephrosis and/or hydroureter caused by POP, even in asymptomatic cases.

All procedures performed in this study were in accordance with the tenets of the 2013 revision of the Declaration of Helsinki and the ethical standards of the National Defense Medical College. This study protocol was accepted on August 21, 2020, by the National Defense Medical College Ethics Committee (Saitama, Japan; ID 4219). Written informed consents were collected from all patients. This study included patients who underwent TVM for POP within the above-mentioned timeframe and excluded those who declined to participate.

The median postoperative observation period was 12.2 months (interquartile range: 9.6-18.2). The median operative time calculated from all patients was 62 min (interquartile range: 54-73).

Surgical methods

**Figure 1:**
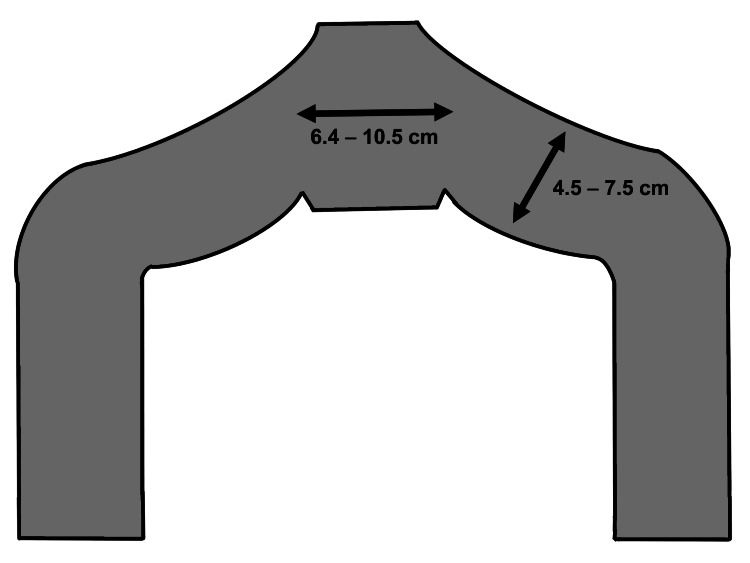
Stencil papers for uphold-type TVM Each mesh arm was withdrawn through the respective skin incision as described in the surgical methods subsection of the materials and methods section. These figures were created by the first author, Kuroda, using Procreate (Savage Interactive Pty, Hobart, Australia). Image credits: Kenji Kuroda.

The TVM (uphold-type) method is described in our previous studies [5,6]. Briefly, the procedure started with a hydrodissection. Subsequently, the anterior vaginal wall was incised vertically, and the pubocervical fascia was fully dissected laterally using a blunt technique to expose the sacrospinous ligaments. Next, a skin incision was made 4 cm laterally and 3 cm inferior to the anal center. Targeting a position of one or two fingerbreadths medial to the ischial spine, a Shimada needle threaded with nylon monofilament sutures was then used to further penetrate the sacrospinous ligaments from the incision. The mesh arms, ORIHIME (Kono Seisakusho, Japan, Tokyo), were then removed with nylon monofilament loops, and an acceptable shape was created, spread, and secured under the bladder. The meshes were cut to fit stencil paper with two arms in advance. The arm width was initially set to 4.5 to 6 cm but later increased to approximately 6 to 7.5 cm (Figure 1). Lastly, traction was used over the externalized arms to ensure proper alignment, and the vaginal wound was closed using 2-0 Vicryl (Johnson and Johnson, Tokyo, Japan) sutures.

Assessment of postoperative recurrence

Prolapse recurrence was defined as the most dependent portion being at POP stage ≥ 2, which means that the most distal prolapse portion is ≥ −1 cm from the hymen plane, according to Takazawa et al. [12].

Statistical analysis

Pearson’s chi-square test was conducted to evaluate the correlation between preoperative plus intraoperative factors and prolapse recurrence. The multiple logistic regression model and the Cox proportional hazards model were used to determine independent factors that contribute to prolapse recurrence. Statistical analysis was performed using JMP PRO version 17 (SAS Institute, Cary, NC). A p-value of < 0.05 was considered statistically significant.

## Results

Table 1 shows the clinical data, such as age, BMI, the presence of diabetes mellitus, POP stage, previous history of hysterectomy, blood loss, operative time, intraoperative complications, mesh arm width, and mode of prolapse recurrence. Intraoperative peritoneal injury was observed in two patients; however, there were no serious postoperative complications noted. Both were rated as Clavien-Dindo classification grade 1 [13].

**Table 1:** Clinical characteristics of the patients TVM: transvaginal mesh surgery. IQR: interquartile range. SD: standard deviation. BMI: body mass index. POP: pelvic organ prolapse

Factors	TVM (n = 104)	
Age, median (IQR)	77 (72 - 81.75)	
BMI (kg/m^2^), mean ± SD	24.7 ± 3.5	n
Diabetes mellitus	Present	21
	Absent	83
POP stage	Stage 3	89
	Stage 4	15
Previous hysterectomy	Present	29
	Absent	75
Blood loss (mL), median (IQR)	25 (15.3 - 42.5)	
Operative time (min), median (IQR)	62 (54 - 73)	
Intraoperative complications	Peritoneal injury	2
Mesh arm width	< 6 cm	32
	≥ 6 cm	72
Recurrent POP	Cystocele	10

Table 2 shows that POP stage 4 and the mesh arm width < 6 cm were significantly associated with prolapse recurrence, according to the correlation analysis between pre- and intraoperative variables and prolapse recurrence (both p < 0.05, Pearson’s chi-square test).

**Table 2:** Association between prolapse recurrence and pre- and intraoperative variables BMI: body mass index. POP: pelvic organ prolapse

Factors	Prolapse recurrence		P value	Chi-square value
Age	Present	Absent		
≥ 77	7 (12.7%)	48 (87.3%)	0.2541	1.301
< 77	3 (6.1%)	46 (93.9%)		
BMI				
≥ 25	5 (10.0%)	45 (90.0%)	0.8981	0.016
< 25	5 (9.3%)	49 (90.7%)		
Diabetes mellitus				
Present	3 (14.3%)	18 (85.7%)	0.4164	0.66
Absent	7 (8.4%)	76 (91.6%)		
POP stage				
Stage 4	4 (26.7%)	11 (73.3%)	0.0155	5.864
Stage 3	6 (6.7%)	93 (93.3%)		
Previous hysterectomy				
Present	5 (17.2%)	24 (82.7%)	0.1009	2.691
Absent	5 (6.7%)	70 (93.3%)		
Mesh arm width				
< 6 cm	8 (25.0%)	24 (75.0%)	0.0004	12.588
≥ 6 cm	2 (2.8%)	70 (97.2%)		

Furthermore, multivariate logistic regression analysis was performed to identify factors showing an independent association with prolapse recurrence. Among the pre- and intraoperative factors (including age, BMI, comorbid diabetes mellitus, POP stage, and prior hysterectomy), only the mesh arm width < 6 cm was a significant predictor of prolapse recurrence (p = 0.0077, multivariate multiple logistic regression analysis) (Table 3).

**Table 3:** Factors contributing to the occurrence of prolapse recurrence in multiple logistic regression analysis OR: odds ratio. CI: confidence interval. BMI: body mass index. POP: pelvic organ prolapse

	Univariate				Multivariate			
Factors	OR	95% CI		P-value	OR	95% CI		P-value
Age (≥ 77 or < 77)	2.24	0.55	9.17	0.2639				
BMI (kg/m^2^) (≥ 25 or < 25)	1.09	0.30	4.01	0.8982				
Diabetes mellitus (Present or Absent)	1.81	0.43	7.69	0.4217				
POP stage (4 or 3)	5.03	1.22	20.66	0.0250	2.83	0.61	13.05	0.1827
Previous hysterectomy (Present or Absent)	2.92	0.78	10.96	0.1129				
Mesh arm width (< 6 cm or ≥ 6 cm)	11.67	2.31	58.80	0.0029	9.48	1.81	49.61	0.0077

According to the univariate Cox proportional hazards model, patients treated with ORIHIME mesh of arm width <6 cm had a significantly higher risk of prolapse recurrence (p = 0.0033). In the multivariate analysis, mesh arm < 6 cm was the only variable showing an independent association with a shorter time to prolapse recurrence (hazard ratio, 8.39; 95% confidence interval, 1.70 - 41.35; p = 0.0089, multivariate Cox proportional hazards analysis) (Table 4).

**Table 4:** Factors contributing to prolapse-recurrence-free survival in the Cox proportional hazard model HR: hazard ratio. CI: confidence interval. BMI: body mass index. POP: pelvic organ prolapse

	Univariate				Multivariate			
Factors	HR	95% CI		P-value	OR	95% CI		P-value
Age (≥ 77 or < 77)	2.16	0.56	8.36	0.2645				
BMI (kg/m^2^) (≥ 25 or < 25)	1.10	0.32	3.82	0.8752				
Diabetes mellitus (Present or Absent)	1.69	0.44	6.55	0.4452				
POP stage (4 or 3)	4.32	1.22	15.31	0.0235	2.35	0.64	8.64	0.1985
Previous hysterectomy (Present or Absent)	2.57	0.74	8.89	0.1354				
Mesh arm width (< 6 cm or ≥ 6 cm)	10.22	2.17	48.24	0.0033	8.39	1.70	41.35	0.0089

**Figure 2:**
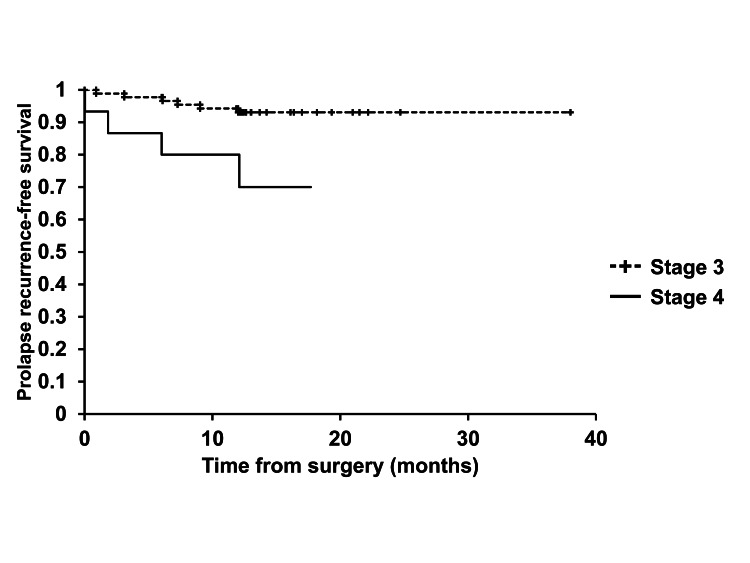
Prolapse recurrence-free survival between patients with stage 3 POP and those with stage 4 POP There was a significant difference in time to prolapse recurrence between patients with stage 3 POP and those with stage 4 POP.

**Figure 3:**
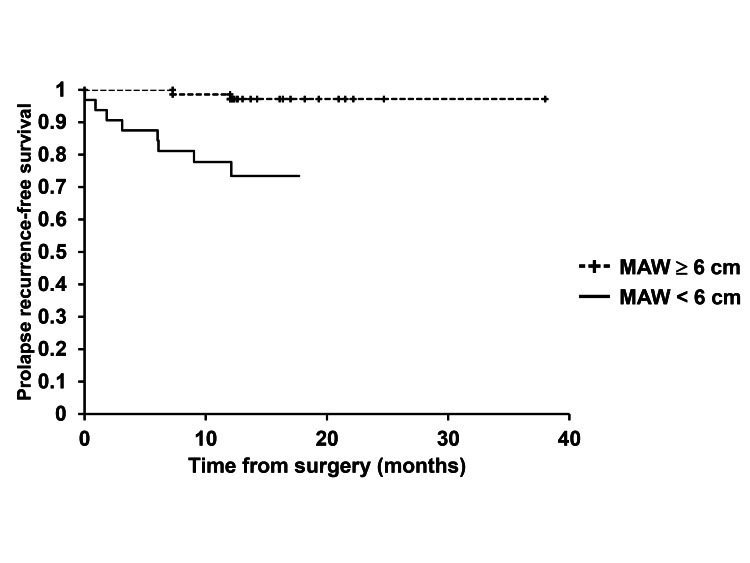
Prolapse recurrence-free survival between patients treated with mesh arm width (MAW) ≥ 6 cm and those treated with MAW < 6 cm There was a significant difference in time to prolapse recurrence between patients treated with mesh arm width (MAW) ≥ 6 cm and those treated with MAW < 6 cm.

In Kaplan-Meier analyses, there were significant differences in prolapse recurrence-free survival rates of patients with stage 4 POP and those with stage 3 POP, as well as between patients treated with ORIHIME mesh of arm width < 6 cm and those treated with ORIHIME mesh of arm width ≥ 6 cm (log-rank test, p = 0.0135, p = 0.0003, respectively) (Figures 2, 3).

## Discussion

In this study, using a mesh with an arm width < 6 cm was significantly associated with prolapse recurrence. Multiple logistic regression analysis and the Cox proportional hazards model showed that only the mesh with arm width < 6 cm was an independent predictor of prolapse recurrence. The prolapse recurrence-free survival rates were significantly lower in patients treated with ORIHIME mesh of arm width < 6 cm compared to those treated with ORIHIME mesh of arm width ≥ 6 cm. The mode of recurrence was cystocele in all recurrent cases.

Because of safety concerns, the US Food and Drug Administration (FDA) has taken regulatory measures regarding TVM for POP repair. Following warnings in 2008 and 2011 about postoperative complications such as mesh exposure, mesh retraction, pain, and dyspareunia [14], the FDA ordered all manufacturers to immediately stop the sales and distribution of transvaginal surgical mesh products for POP repair in the USA in 2019.

In April 2019, Japan’s regulatory authorities banned the use of Polyform™ (Boston Scientific Japan, Tokyo, Japan) PP mesh for TVM procedures because of escalating reports of mesh-related postoperative complications in countries outside Japan. Concurrently, the PTFE mesh ORIHIME received approval in May 2019. Since then, ORIHIME has been used in all TVM cases at our institution. Japanese TVM outcomes have been marked by low POP recurrence and mesh-related complication rates [15,16], as well as significant quality-of-life enhancements for patients [12,17,18]. Our experience with TVM using ORIHIME mesh has been particularly positive, with no severe postoperative complications observed in our previous studies [5,6].

Several studies have found higher recurrence rates after TVM using ORIHIME compared to TVM using PP [19,20]. In one study, recurrence in the operated compartment was significantly more common in patients treated with TVM using ORIHIME. As of the four-year follow-up, three cases (9.1%) treated with TVM using PP mesh and 10 cases (33.3%) treated with TVM using ORIHIME showed recurrence [19]. In another study, 10 out of 104 patients who underwent PP mesh placement showed recurrence (9.6%) compared to nine out of 67 patients who underwent ORIHIME placement (13.4%) after one-year follow-up [20]. In animal studies using rats, the inflammation scores based on histological analysis and collagen morphometry were lower in groups treated with PTFE mesh as well as other types of mesh [21], and other mesh-related complications such as mesh exposure were less common [11]. Because of its lower tissue adherence and minimal inflammatory response compared to PP mesh, PTFE mesh is expected to pose challenges in maintaining its positioning [11,19].

Our experience has shown that increasing the mesh arm width can reduce the recurrence rate. Indeed, in our previous study, the rate of POP recurrence after TVM using ORIHIME was significantly lower, 2.9% (1/35), in the group with arm width ≥ 6 cm compared to the group with arm width < 6 cm (2.9% [1/35] vs. 25% [6/24], respectively) [5]. Moreover, in another study, the incidence of urinary incontinence was lower in patients treated with TVM using ORIHIME with wider arms and adjusted length compared to those treated with ORIHIME with wider arms only [6]. These findings underline the importance of the mesh shape, including arm width, in preventing the sliding off of the mesh from the sacrospinous ligament, given the expected low coefficient of friction of ORIHIME.

Some limitations of this study should be acknowledged. First, this was a single-center retrospective study with a relatively small sample size. Second, the median follow-up duration following surgery was rather short. Larger multicenter studies with longer follow-up duration are required to obtain more robust evidence. Despite these limitations, we believe that our findings provide the rationale for continuing to implement TVM.

## Conclusions

Narrow mesh arm width can contribute to postoperative POP recurrence after TVM using ORIHIME because of the low expected coefficient of friction. TVM using wide-arm OIHIME was associated with lower recurrence rates compared to TVM using narrow-arm ORIHIME. This suggests that making wide arm adjustments may be a better treatment strategy when performing TVM using ORIHIME. Patients with stage 4 POP should also be carefully monitored postoperatively. 
